# Multi-level optimal energy management strategy for a grid tied microgrid considering uncertainty in weather conditions and load

**DOI:** 10.1038/s41598-024-59655-7

**Published:** 2024-05-02

**Authors:** H. E. Keshta, E. G. Hassaballah, A. A. Ali, K. M. Abdel-Latif

**Affiliations:** 1 Faculty of Engineering at Shoubra, Benha University, Banha, Egypt; 2 Faculty of Engineering, Helwan University, Cairo, Egypt; 3 Greater Cairo Water Company, Cairo, Egypt

**Keywords:** Engineering, Electrical and electronic engineering

## Abstract

Microgrids require efficient energy management systems to optimize the operation of microgrid sources and achieve economic efficiency. Bi-level energy management model is proposed in this paper to minimize the operational cost of a grid-tied microgrid under load variations and uncertainties in renewable sources while satisfying the various technical constraints. The first level is day ahead scheduling of generation units based on day ahead forecasting of renewable energy sources and load demand. In this paper, a recent meta-heuristic algorithm called Coronavirus Herd Immunity Optimizer (CHIO) is used to solve the problem of day-ahead scheduling of batteries, which is a complex constrained non-linear optimization problem, while the Lagrange multiplier method is used to determine the set-point of the Diesel Generator (DG). The second level of the proposed EMS is rescheduling and updating the set-points of sources in real-time according to the actual solar irradiance, wind speed, load, and grid tariff. In this paper, a novel real-time strategy is proposed to keep the economic operation during real-time under uncertainties. The obtained results show that the CHIO-based bi-level EMS demonstrates an optimal economic operation for a grid-connected microgrid in real-time when there are uncertainties in weather, utility tariffs, and load forecasts.

## Introduction

Microgrid (MG) is a small-scale electrical grid that consist of Distributed Energy Resources (DERs) such as Photovoltaics (PVs), Wind Turbines (WTs), and Diesel Generators (DGs), energy storage devices like batteries and super capacitors, and loads^1^. The operation of the MG is divided into two modes: islanded mode and grid-connected mode^2^. In grid connected mode, the MG keeps power balance between supply and demand by exchanging power with the main grid, buying power from or selling power to the main grid. MGs are more efficient than conventional centralized thermal power plants because they can reduce power loss and voltage drop associated with long-distance transmission, and carbon emissions resulting from conventional power plants. Furthermore, the MG can achieve high energy independence because it can be operated independently of existing power systems^3^.

An effective energy management strategy (EMS) is necessary for a microgrid system to operate economically^4^. It should schedule DERs, storage devices, power exchange with the main grid, and controllable loads optimally based on historical and current data while meeting various technical constraints^5^. The EMS manages the flow of power within the MG through providing reference profiles for the MG's controllers based on predetermined objectives^6^. The presented paper introduces an efficient strategy for energy management and minimize the daily operating cost of a grid-connected MG based on two levels: optimal day-ahead scheduling based on day ahead forecasting and real-time scheduling.

Day-ahead Predictions of load, market prices of electricity, and renewable energy sources (RESs) are used in energy management systems to schedule the output power of each generation unit in the next day optimally^7^. However, the inaccurate predictions of RESs, loads, and market prices may lead to high uncertainties in sources scheduling during real-time. For accurate predictions, day-ahead energy management methods that take into account statistical data are typically used^8,9^. Support vector machine regression based load forecasting model was applied in^10^. In^11^, short term load demand forecasting based on five families of regression models was discussed using MATLAB Regression Toolbox. In this paper, artificial neural network (ANN) based day-ahead forecasting is proposed to accurately predict future variables such as solar irradiance, wind speed and load demand. It is compared with the forecasting techniques that are based on support vector machine and conventional regression to verify its performance. The real-time EMS is required to deal with various uncertainties caused by prediction errors of renewable generation, load, and market price. The real-time scheduling is used not only to reduce overall operating costs but also to guarantee the stability of the microgrid^12^. However, a large number of earlier studies on microgrid energy management neglect to account for real-time economic dispatch. In^5^, day-ahead scheduling of microgrid units using Mixed-Integer Linear Programming (MILP) was presented, ignoring forecasts of load, weather, and grid tariff uncertainties. Day ahead scheduling of AC/DC hybrid microgrid was presented in^13^ taking into account only grid tariff uncertainties while neglecting real-time scheduling. The economic rescheduling of battery set-points during the real-time operation of networked multi-microgrids was not taken into consideration in^14^. Furthermore, it was neglected in^15–17^ to modify the operating points of MG sources in order to maintain economic operation in the face of weather-related and load-demand disturbances. A day-ahead scheduling approach based on a stochastic optimization model was presented in^18,19^, but it was unable to achieve real-time economic operation since the MG sources' set-points were not updated in accordance with the actual system conditions during real-time operation. In^20^, a day-ahead scheduling using an enhanced grey wolf optimizer for a grid-connected microgrid was proposed; however, real-time operation uncertainties were not taken into account. The online EMS was used in^21^ for grid-tied microgrids establishes the optimal operating points for sources at each hour independently of the other hours of the day. As a result, it might not offer the best operation when taking into account the overall cost of daily operation. Furthermore, the set-points of the sources in a grid-connected MG were calculated at daily intervals^22^. Although some research has been conducted on the energy management of microgrids, there is still a research gap for the energy management of microgrids during real-time operation. This paper presents a novel real-time energy management approach based on ANN to update the operating points of the batteries and DG in the MG during real-time operation using actual weather, grid tariff, and load demand in order to achieve the economic operation under uncertainties.

Many optimization algorithms are presented in the literature to improve optimal control approaches for battery energy. In recent times, a number of advanced nature-inspired meta-heuristic algorithms have been proposed to effectively handle and solve complicated micro-grid optimization problems, exceeding the capabilities of traditional deterministic methods. The dolphin echolocation algorithm (DEA) was applied in^23^ for the scheduling of RESs in the micro-grid. In^24^, an optimal scheduling of the power generation in the micro-grid including some RESs, was provided based on the memory based genetic algorithm (MGA). The modified particle swarm optimization algorithm was applied in^25^ for scheduling renewable generation in a micro-grid under load uncertainty. A multi-objective scheduling problem of MGs was solved utilizing the teaching learning based optimization algorithm^26^. Coronavirus herd immunity optimizer (CHIO) is a state-of-the-art optimization algorithm that is superior to other metaheuristic techniques in finding the global minimum value of 24 standard benchmark functions^27^. In this paper, the optimization problem of the day-ahead scheduling problem is solved by using CHIO.

The major contributions of the paper are: (i) Introducing an efficient bi-level EMS that includes day-ahead scheduling and real-time scheduling, (ii) Proposing an artificial intelligence based forecasting model to predict the load, solar irradiance and wind speed of the next day. The performance of the proposed artificial intelligence based approach is also compared with forecasting techniques that are based on support vector machine and the regression based approach (a traditional technique) to evaluate its effectiveness, (iii) presenting an ANN based real-time EMS that reschedules and updates the optimal set-setpoints of sources and batteries within the MG to attain the economic operation under the real-time uncertainty of weather conditions, load demand and electricity tariff, (iv) Applying an advanced meta-heuristic technique, CHIO, to solve the day-ahead scheduling problem.

The rest of the paper is ordered as: In the subsequent section, the system under investigation is described, and the modeling of its components is presented. The suggested bi-level EMS and the used recent optimization algorithm (CHIO) are introduced in “Bi-level energy management strategy” section. “Simulation results” section provides an analysis and discussion of the simulation results obtained. Finally, the last section presents the conclusion derived from the results.

## The proposed microgrid structure

**Figure 1:**
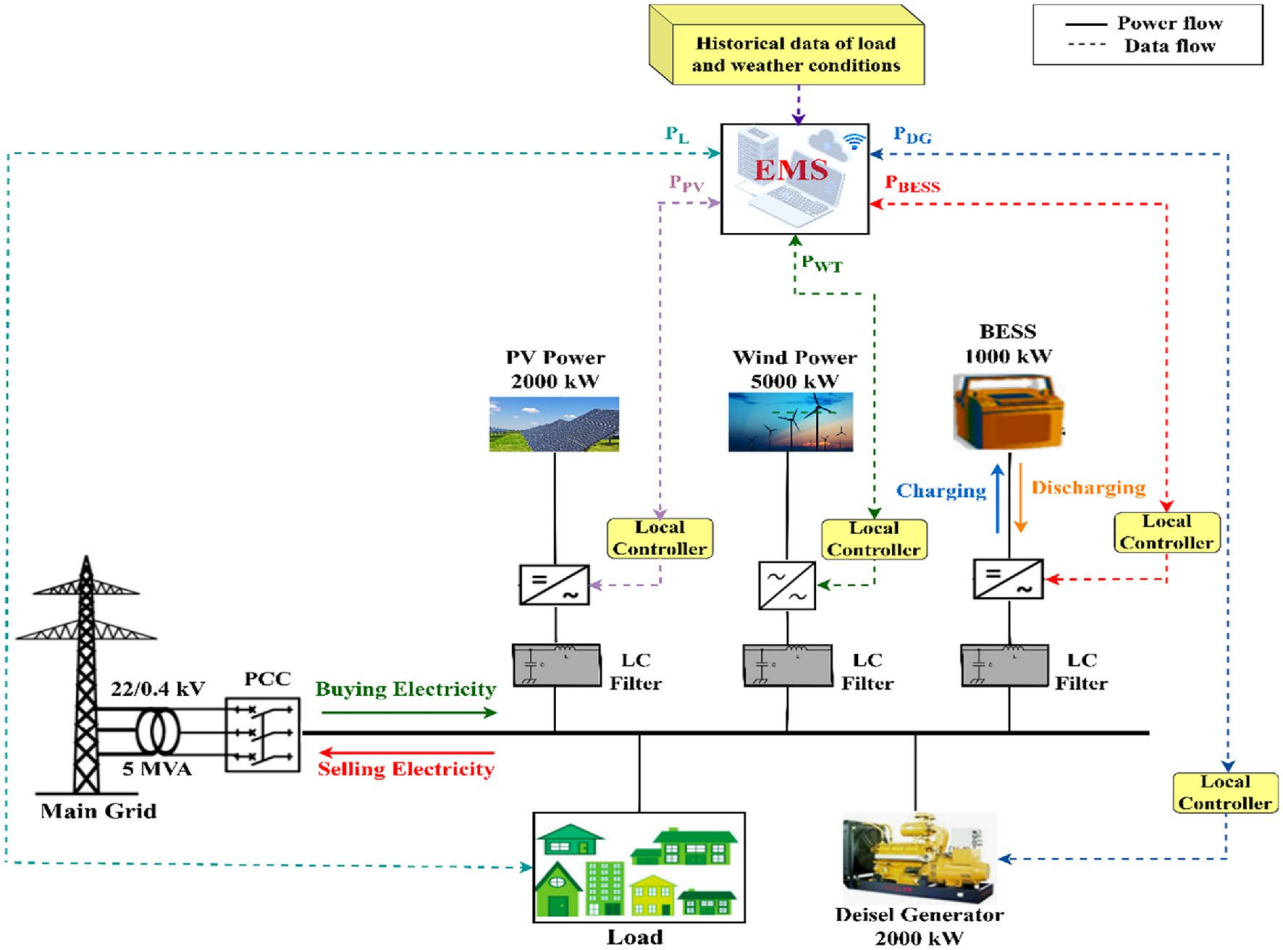
The scheme of the system under study.

As shown in Fig. 1, the grid-tied microgrid system under consideration consists of a 2000 kW solar power plant, a 5000 kW wind farm, a 2000 kW DG, and 4000 kWh lithium-ion batteries. The solar power plant has four thousand 500 W PV modules, and the wind farm has ten 500 kW wind turbines. An inverter connects the DC output of PV to an AC bus. The WT is connected to the AC bus through an AC/AC converter, while the BESS is connected through a bidirectional converter. Each MG unit operates in its own manner. The battery, for example, has two operating modes: charging and discharging, whereas the WT or PV source can operate in either limiting power or maximum power point tracking mode. As a result, each source in the microgrid has a local controller. The central controller is responsible for executing the EMS of the MG, where it communicates with the various sources and loads within the MG and makes decisions that result in the most economical operation possible. The input variables of the central controller are solar irradiance, wind speed, grid tariff, and load in real time, as well as the operating state of sources and load connected to the MG. The central controller sends commands directly to the local controller, which in turn controls the converter interfaced source to maintain its power at a predetermined level. The proposed EMS in this paper will be discussed in greater detail in the following section. The system parameters are listed in the appendix.

An important first step in energy management is precise modelling, because the optimization algorithm uses it in order to determine the optimal dispatch decisions. Distributed energy sources in the MG and their related constraints are modelled in the following subsections.

### PV model

The generated power by photovoltaic array at time t is a function of the incident solar radiation (G_in_), and the cell temperature (T_C_) at time t as shown in Eq. (1)^28^.

1 $${\text{P}}_{\text{PV}}({\text{t}}) =\mathrm{ A_{P} }\times \mathrm{ N_{PV} }\times \mathrm{ G_{in} }({\text{t}}) \times (1-\mathrm{\alpha }(\mathrm{T_{C} }({\text{t}})-\mathrm{ T_{r}})) \times {\eta }_{m}\times {\eta }_{convS}$$

where: A_P_ is the area of PV modules (m^2^), N_PV_ is the number of PV modules in the PV array, ${\eta }_{m}$ represents Module efficiency and ${\eta }_{convS}$ represents the DC/AC converter efficiency of solar, and T_r_ is the reference temperature (T_r_ = 25 ℃)***.***

### WT model

The output power of a wind farm, which consists of N_w_ wind turbines at any given time t (P_WT_(t)), is proportional to the prevailing wind speed (V_t_) and can be calculated, as shown in Eq. (2). The wind speed is measured over a 24-h period, taking into account the minimum wind speed required to start the turbine (cut-in wind speed) and the maximum wind speed required to stop the turbine from running (cut-out wind speed)^28^:

2 $${\text{P}}_{\text{WT}}({\text{t}}) =\mathrm{ N_{W} }\times {\eta }_{c{\varvec{o}}{\varvec{n}}{\varvec{v}}{\varvec{W}}}\times \left\{ {\begin{array}{*{20}ll}    0 & \quad {{\text{V}}_{{{\text{co}}}}  < {\text{V}}_{{\text{t}}}  < {\text{V}}_{{{\text{ci}}}} }  \\    {{\text{a}}*\left( {{\text{V}}_{{\text{t}}} } \right)^{3}  - {\text{b}}*{\text{P}}_{{{\text{nom}}}} } & \quad {{\text{V}}_{{{\text{ci}}}}  < {\text{V}}_{{\text{t}}}  < {\text{V}}_{{\text{r}}} }  \\    {{\text{P}}_{{{\text{nom}}}} } & \quad {{\text{V}}_{{\text{r}}}  < {\text{V}}_{{\text{t}}}  < {\text{V}}_{{{\text{co}}}} }  \\ \end{array} } \right. $$

where: V_r_, P_nom_, ${\eta }_{c{\varvec{o}}{\varvec{n}}{\varvec{v}}{\varvec{W}}}$, V_co_ and V_ci_ refer to the rated wind speed, rated power of the WT, the AC/AC converter efficiency, cut-out wind speed and cut-in wind speed, respectively. a and b are wind coefficients and can be determined as shown in the following equations.

3 $$a=\frac{{{\text{P}}}_{{\text{nom}}}}{{\left({{\text{V}}}_{{\text{r}}}\right)}^{3}-{\left({{\text{V}}}_{{\text{ci}}}\right)}^{3}}$$

4 $$b=\frac{{\left({{\text{V}}}_{{\text{ci}}}\right)}^{3}}{{\left({{\text{V}}}_{{\text{r}}}\right)}^{3}-{\left({{\text{V}}}_{{\text{ci}}}\right)}^{3}}$$

### DG model

The operational cost of DG at time t (C_DG_ (t)) can be expressed as a quadratic function of its output power as follows^29^:

5 $$\mathrm{C_{DG} }({\text{t}}) = \left({\mathrm{\alpha }}_{{\text{DG}}}+{\upbeta }_{{\text{DG}}}  \times {{\text{P}}}_{{\text{DG}}}\left({\text{t}}\right)+{\upgamma }_{{\text{DG}}} \times {{\text{P}}}_{{\text{DG}}}^{2}({\text{t}})\right)$$

where $\mathrm{\alpha }$_DG_, $\upbeta$_DG_, and γ_DG_ are the fuel cost coefficients of DG.

The power delivered by the DG at time t (P_DG_(t)) shall be limited between minimum ${{\text{P}}}_{{\text{DG}},{\text{min}}}$ and maximum ${{\text{P}}}_{{\text{DG}},{\text{max}}}$, as shown in the following equation:

6 $${{\text{P}}}_{{\text{DG}}}^{{\text{min}}}\le {{\text{P}}}_{{\text{DG}}}\left({\text{t}}\right)\le {{\text{P}}}_{{\text{DG}}}^{{\text{max}}}$$

### Battery energy storage system (BESS) model

The provided power by battery at time t (P_BESS_(t)) shall be bounded between its minimum and maximum limits as:

7 $${{-{\text{P}}}_{{\text{BESS}}}^{{\text{max}}}\le {\text{P}}}_{{\text{BESS}}}\left({\text{t}}\right)\le {{\text{P}}}_{{\text{BESS}}}^{{\text{max}}}$$

The stored energy in the battery at time t (E(t)) and the corresponding state of charge (SOC(t)) can be computed as follows^30^:

8 $${\text{E}}\left({\text{t}}\right)=\left\{\begin{array}{l}E\left({\text{t}}-1\right)-\frac{\Delta \mathrm{T X}{\mathrm{ P}}_{{\text{BESS}}}\left({\text{t}}\right)}{{\upeta }_{{\text{dis}}}} ,{{\text{P}}}_{{\text{BESS}}}\left({\text{t}}\right)>0\\ E\left({\text{t}}-1\right)-\Delta T{ \times\upeta }_{\mathrm{ch }} \times {{\text{P}}}_{{\text{BESS}}}\left({\text{t}}\right) ,{{\text{P}}}_{{\text{BESS}}}\left({\text{t}}\right)\le 0\end{array}\right.$$

9 $${\text{SOC}}\left({\text{t}}\right)=\frac{{\text{E}}\left({\text{t}}\right)}{{{\text{B}}}_{{\text{Capacity}}}}$$

where: ${\eta }_{c{\text{h}}}$ and ${\eta }_{dis}$ represent the battery efficiency during charging and discharging mode, respectively. $\Delta {\text{T}}$ is sampling time (1 h) and B_capacity_ represents the battery capacity in kWh.

The inequality in Eq. (10) ensures that the SOC of the battery is within the minimum and maximum limits of the SOC.

10 $${{\text{SOC}}}_{{\text{min}}}\le {\text{SOC}}\left({\text{t}}\right)\le {{\text{SOC}}}_{{\text{max}}}$$

The operating cost of BESS at time t (C_BESS_(t)), considering the degradation cost due to fast charging and discharging, can be calculated as follows^31^:

11 $$\mathrm{C_{BESS} }({\text{t}}) =\frac{{{\text{CC}}}_{{\text{bat}}}{\mathrm{ x \eta }}_{{\text{ch}}}\mathrm{x }\Delta \mathrm{T x }{{\text{P}}}_{{\text{BESS}},{\text{ch}}}\left({\text{t}}\right)}{2  \times {{\text{N}}}_{{\text{cycle}}}}+\frac{{{\text{CC}}}_{{\text{bat}}} \times \Delta \mathrm{T x }{{\text{P}}}_{{\text{BESS}},{\text{dis}}}\left({\text{t}}\right)}{{\upeta }_{\mathrm{dis }}{\text{x}}2 \times {{\text{N}}}_{{\text{cycle}}}}+{\mathrm{ C}}_{{\text{deg}}} \times {\mathrm{ P}}_{\mathrm{BESS }}(t) $$

where P_BESS_ (t) is the net power produced by the battery (P_BESS,dis_ – P_BESS,ch_), CC_bat_ is the capital cost of the battery, N_cycle_ is the number of battery life cycles and C_deg_ is a factor to penalize the high stress during the charging and discharging process that causes the batteries to deteriorate.

### Main grid model

The tie-line exchange power must remain within the allowable limits (${{\text{P}}}_{{\text{grid}}}^{{\text{min}}}$ and ${{\text{P}}}_{{\text{grid}}}^{{\text{max}}}$) as follows:

12 $${{\text{P}}}_{{\text{grid}}}^{{\text{min}}}\le {{\text{P}}}_{{\text{grid}}}\left({\text{t}}\right)\le {{\text{P}}}_{{\text{grid}}}^{{\text{max}}}$$

When P_grid_ is positive or negative, it indicates that the MG is importing or exporting active power to or from the main grid.

The total active power balance equation in the MG is calculated as follows:

13 $$ {\text{P}}_{{{\text{grid}}}} \left( {\text{t}} \right) \, = {\text{ P}}_{{\text{L}}} \left( {\text{t}} \right) \, {-}{\text{P}}_{{{\text{PV}}}} \left( {\text{t}} \right) \, {-}{\text{P}}_{{{\text{WT}}}} \left( {\text{t}} \right) \, {-}{\text{ P}}_{{{\text{BESS}}}} \left( {\text{t}} \right) \, - {\text{P}}_{{{\text{DG}}}} \left( {\text{t}} \right) $$

where P_L_ is the predicted load at time t.

## Bi-level energy management strategy

The proposed EMS aims to achieve the optimal power sharing between DG, BESS and the grid to meet the system load demand and achieve MG economic dispatch. The objective function of the suggested EMS is to minimize the microgrid's daily operating cost (C_OP_MG_), and it is formulated as follows:

14 $$ {\mathrm{Min\;\;C}}_{{{\text{OP\_MG}}}}  =\sum_{{\text{t}}=1}^{24}( {\text{C}}_{{{\text{MG-buy}}}}  + {\text{C}}_{{{\text{MG-sell}}}}  +\mathrm{ C_{BESS} }+\mathrm{ C_{DG}})$$

where C_MG-buy_ and C_MG-sell_ are the MG purchase and sale prices ($) of electricity from and to the main electrical grid, respectively, and can be computed as follows:

15 $$ {\text{C}}_{{{\text{MG-buy}}}}  = {{\text{C}}}_{{\text{grid-buy}}} \left({\text{t}}\right) \times {{\text{P}}}_{{\text{grid}}}\left({\text{t}}\right), \quad {{\text{P}}}_{{\text{grid}}}\left({\text{t}}\right) > 0$$

16 $${\text{C}}_{{{\text{MG-sell}}}}= {{\text{C}}}_{{\text{grid-sell}}} \left({\text{t}}\right) \times {{\text{P}}}_{{\text{grid}}}\left({\text{t}}\right), \quad {{\text{P}}}_{{\text{grid}}}\left({\text{t}}\right)<0$$

The buying and selling energy tariffs,C_grid-buy_ and C_grid-sell_, are illustrated in Table 1.

**Table 1:** Purchasing and selling electricity tariffs^6^.

Type	Time	Value
Off-peak purchasing tariff	From 12 to 7 a.m.	0.06 $/kWh
Mid-peak purchasing tariff	From 7 a.m. to 4 p.m.	0.144 $/kWh
Peak purchasing tariff	From 4 to 8 p.m.	0.252 $/kWh
Mid-peak purchasing tariff	From 8 p.m. to 12 a.m.	0.144 $/kWh
Fixed selling tariff	All day	0.0582 $/kWh

**Figure 2:**
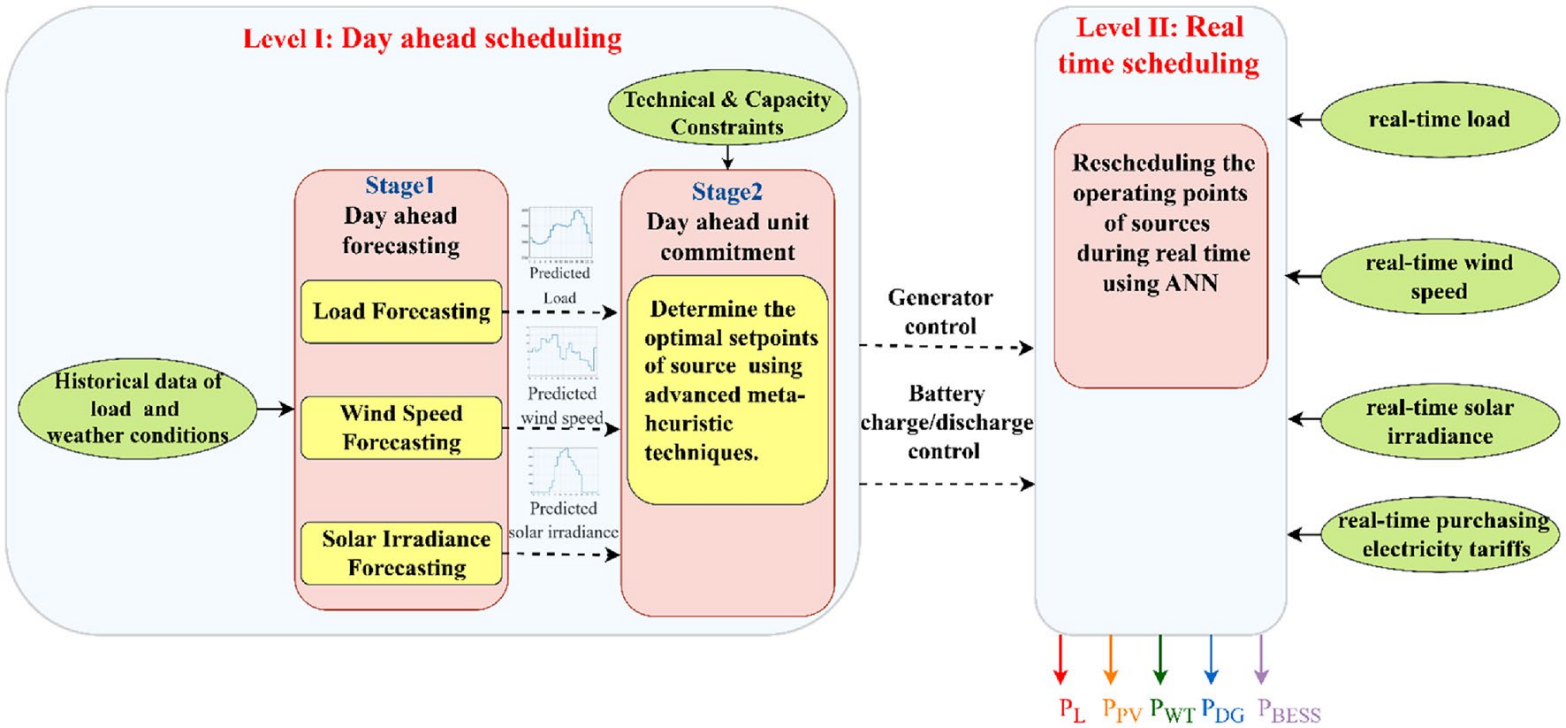
The proposed energy management strategy.

The proposed bi-level EMS operates on two levels (day-ahead scheduling and real-time scheduling), with the aim of meeting the load demand of MG at the lowest possible operating cost based on predicted data for the next 24 h and real data. The flow chart of the proposed EMS is shown in Fig. 2.

### Level I: Day-ahead scheduling

This level of EMS is divided into two stages; the first stage is day-ahead forecasting of solar irradiance, wind speed, and load demand, while the other stage is day-ahead unit commitment.

#### Day-ahead forecasting (stage I)

Forecasting is a technique that makes predictions about the future based on past performance and current patterns from a number of inputs. Short term forecasting, day ahead forecasting, for the load and renewable energy sources is applied in this paper. Accurate prediction of renewable energy sources and load helps in providing an optimum energy management system. Artificial neural network (ANN) is applied in this paper for forecasting the load, solar irradiance and wind speed in the next day based on historical data. Also, a comparative performance study between three different forecasting models, the proposed ANN based forecasting, support vector machine based forecasting and the traditional technique known as regression-based forecasting, is investigated in this paper.

##### Regression based approach

Regression is one of the most widely used statistical techniques for finding a relationship between two or more variables^32^.

The model of load forecasting based on regression can be summarized in the following steps:

Step 1: Input data: There are three types of datasets. The first dataset is historical load demand data such as D-1 load (the load of the previous day at the same hour), D-7 load (the load of the previous week at the same day and the same hour), and H-1 Load (the load of the previous hour). The second dataset is historical meteorological data, such as temperature (dry bulb temperature and dew point temperature). The third dataset is time indicators. Time must be taken into account because its impact on the customer's load demand is greatest. Several time markers were used in this study, including the hour, the day, and the holiday indicator (1 for a working day and 0 for a holiday).
Step 2: The regression models will be trained: A model is initially trained using a training dataset, and the results are then examined by changing the model's parameters until the most effective parameters are found.
Step 3: The regression models will be tested: The trained model is then tested using testing datasets, and the performance is assessed using a variety of statistical error indices and evaluation plots after the results are satisfied. The load forecasting model's control parameters are optimized using testing data, which is hidden from the trained model and used to assess and improve the performance of the developed model.
Step 4: The forecast load is compared to the actual measured load, and statistical error matrices are used to measure how accurate the forecast is.

##### Support vector machine (SVM)

Support Vector Machines (SVM) are a type of machine learning technique that was developed to address non-linear classification problems. Support vector regression (SVR), the most popular type of SVM, is specifically made to address regression issues. The main objective of SVR is to create a model that uses known inputs to predict unknown outputs^33^. The input data for Support Vector Machines (SVM) of load forecasting is 7 variables: the hour, the day, the holiday indicator (1 for a working day and 0 for a holiday), temperature (dry bulb temperature & dew point temperature), the load of the previous day at the same hour, the load of the previous week at the same day and the same hour and the load of the previous hour. The data is divided into sets for training and validation, and then the SVM regression technique is used to train the model. To maximize the accuracy and minimize the mean error, various kernel models are trained using a range of cost, gamma, and Mean Square Error (MSE) values. The test data set is used to evaluate the trained model, and then the trained model is tested with random data to evaluate its performance.

##### Artificial intelligence-based techniques (artificial neural networks (ANN))

**Figure 3:**
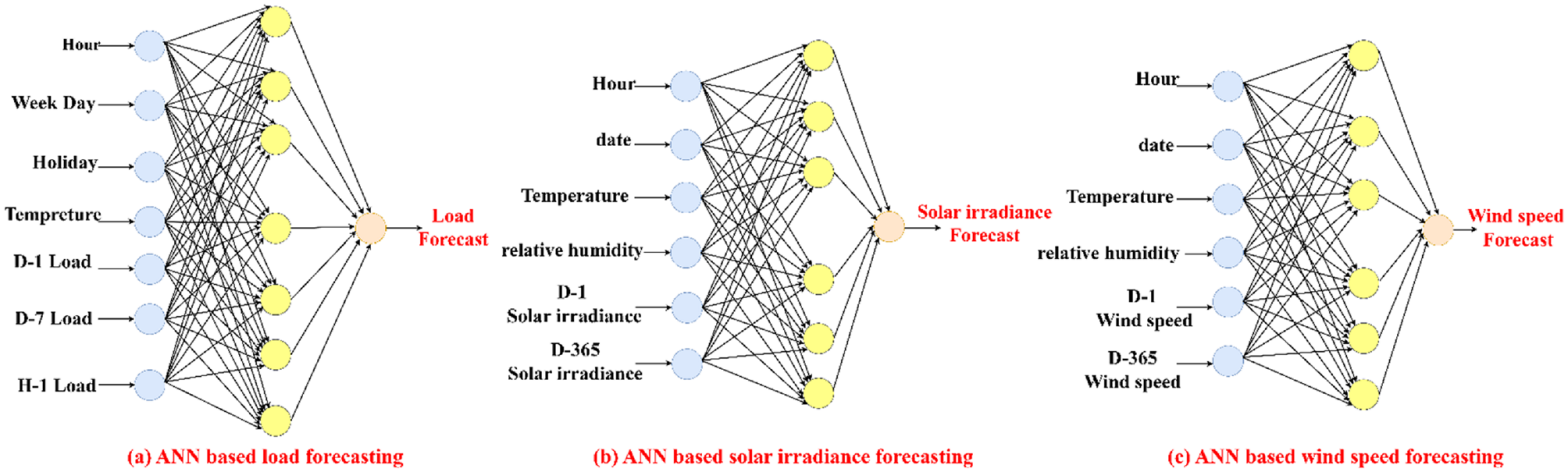
ANN based forecasting models.

ANN, a type of machine learning, is non-linear mathematical processing network that mimics the human brain^34^. The used neural network for load forecasting model consists of an input layer that has 7 neurons, one hidden layer has 100 neurons, and an output layer has one neuron. The input data for the neural network of load forecasting is 7 variables: the hour, the day, the holiday indicator (1 for a working day and 0 for a holiday), temperature (dry bulb temperature and dew point temperature), D-1 load (the load of the previous day at the same hour), D-7 load (the load of the previous week at the same day and the same hour) and H-1 Load (the load of the previous hour) as is clear from Fig. 3a. The output from the neural network is the predicted load in the next day. The input data for ANN based solar irradiance forecasting model is 6 variables: time (hour), date, temperature, relative humidity, D-1 solar irradiance (the solar irradiance of the previous day at the same hour) and D-365 solar irradiance (the solar irradiance of the previous year at the same date and hour) as is clear from Fig. 3b. The output from the neural network is the predicted solar irradiance in the next day. Also, the input data for ANN based wind speed forecasting model is 6 variables: time (hour), date, temperature, relative humidity, D-1 wind speed (the wind speed of the previous day at the same hour and D-365 wind speed (the wind speed of the previous year at the same date and hour) as is clear from Fig. 3c. The output from the neural network is the predicted wind speed in the next day. The neural network is trained by minimizing the cost function, which is typically a quadratic function of output error.

To verify the performance of ANN based forecasting model, it is compared with the forecasting techniques that are based on support vector machine (SVM) and conventional regression.

In this paper, two performance indices, mean absolute percentage error (MAPE) and root mean square error (RMSE), are used to evaluate the forecasting accuracy and can be calculated as:

17 $${\text{MAPE}}= \left(\frac{\left|\mathrm{Forecast \; value }-\mathrm{ true\; value}\right|}{\mathrm{true \; value}}\right) \times 100$$

18 $${\text{RMSE}}=\sqrt{\frac{{\sum }_{1}^{24}{\left(\mathrm{Forecast \; value }-\mathrm{ true \; value}\right)}^{2}}{24}}$$

Lower values for MAPE and RMSE indicate more accurate predictions. The true value of solar irradiance is very small or zero at night, and thus the MAPE for solar irradiance will be infinity or close to infinity^35^. As a result, the MAPE isn't calculated for the solar irradiance forecast. The microgrid proposed by this study is located in the Ras Gharib, Egypt. The historical data is used to train and test the forecasting models. The historical data of weather conditions such as temperature, solar irradiance and wind speed is taken from^36^, while the historical data of load is taken from^37^.

#### Day-ahead unit commitment (stage II)

The power references of sources in the MG are scheduled for the next day based on the day-ahead forecasting of load and output power from PV and WT. The optimal set-points of batteries in the microgrid are obtained by utilizing an advanced meta-heuristic algorithm called CHIO, while the optimal set-point of the DG is determined by using Lagrange multiplier.

CHIO is a nature-inspired human-based optimization algorithm. The inspiration of CHIO is based on the idea of herd immunity and was developed to combat the COVID-19 coronavirus pandemic. The degree to which infected people come into direct contact with other members of society determines how quickly the infection spreads. Social distance is advised by health professionals as a way to safeguard other members of society from the disease. When the majority of the population is immune, a population is said to have reached a state of herd immunity, which prevents the spread of disease. These ideas are modelled using ideas from optimization. CHIO imitates both the ideas of social distance and herd immunity. CHIO has two control parameters and four algorithmic parameters. The two main control parameters are the basic reproduction rate (B_Rr_), which regulates the rate at which the virus pandemic spreads from person to person, and the maximum age (Max__age_) at which a patient can be infected are the control parameters. The main advantages of the CHIO algorithm are having few parameters to be set and its ability to cover the entire search space and escape from local optima by utilizing stochastic-based components, while considering the exploration–exploitation trade-off.

The mathematical model of the CHIO can be summarized in the following steps^27^:

Step 1: Set the parameters for both the optimization problem and the algorithm for optimization.
Step 2: create the initial herd immunity population.
Step 3: According to the percentage of BRr, coronavirus herd immunity has evolved as follows:
  **If** r ≥ BRr (where r produces a number generator between 0 and 1)
  The gene stays the same and doesn't change.
  19 $${g}_{i}^{j}({\text{k}}+1) = {g}_{i}^{j}(k)$$
  where random is a number between 0 and 1 and k is the iteration number.
  **else if** r $<\frac{1}{3}{BR}_{r}$ (infected)
  The gene is updated using the following equation:
  20 $${g}_{i}^{j}({\text{k}}+1) = {g}_{i}^{j}\left(k\right)+\mathrm{ rand}\times ({g}_{i}^{j}\left(k\right)-{g}_{i}^{c}\left(k\right))$$
  where ${{\text{g}}}_{{\text{i}}}^{{\text{c}}}$ is picked at random from any infected situation.
  **else if** r $<\frac{2}{3}{BR}_{r}$ (susceptible)
  21 $${g}_{i}^{j}(k+1) = g\left(k\right)+\mathrm{ rand}\times ({g}_{i}^{j}\left(k\right)-{g}_{i}^{m}\left(k\right))$$
  where ${{\text{g}}}_{{\text{i}}}^{{\text{m}}}$ is chosen at random from any susceptible situation.
  **else** (immune)
  22 $${{\text{g}}}_{{\text{i}}}^{{\text{j}}}({\text{k}}+1) ={{\text{g}}}_{{\text{i}}}^{{\text{j}}}\left({\text{k}}\right)+\mathrm{ r}\times ({{\text{g}}}_{{\text{i}}}^{{\text{j}}}\left({\text{k}}\right)-{{\text{g}}}_{{\text{i}}}^{{\text{V}}}\left({\text{k}}\right))$$
  where ${{\text{g}}}_{{\text{i}}}^{{\text{V}}}$ is the best immune case to date.
  **end**
Step 4: If the generated case has a higher fitness value than the existing one, the herd immunity population matrix (HIM) is updated by replacing it.
Step 5: The infected case would die if its immunity could not increase for a set number of rounds, as indicated by the parameter Max_age.
Step 6: If the termination condition is met, stop after examining the stopping criterion; otherwise, move on to step 3.

**Figure 4:**
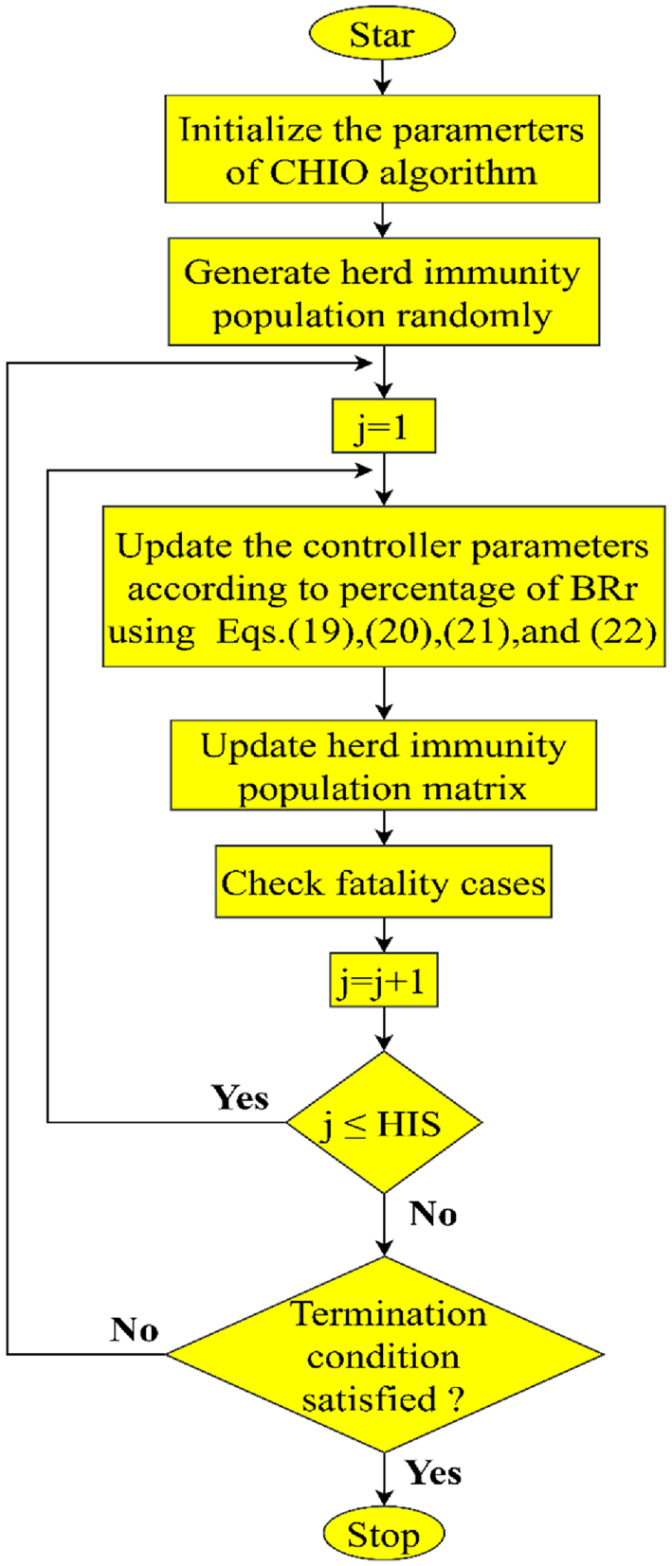
Flowchart of CHIO.

The flowchart that describes the concise summary of the key CHIO steps is depicted in Fig. 4.

### Level II: Real-time scheduling

There is always a percentage error and uncertainty in forecasting of weather conditions and load. The uncertainty of wind speed, solar irradiance, and electricity tariff in this paper is not more than 10%. The microgrid load is dependent on user behavior, whereby electrical devices are operated randomly and unplanned throughout the day. As a result, there may be a significant error between the forecasted and actual demand load, with an actual demand load uncertainty of up to 20%. A real-time EMS that reschedules the optimal operating points of sources during real-time operation is suggested to keep the system balance and cope with the uncertainties of renewable sources, electricity tariff, and load demand in order to achieve the economic operation in real-time.

ANN is utilized in this stage to find the new optimal set-points of batteries in the microgrid, while the Lagrange multiplier method is used to determine the new set-point of the DG.

The new set-point of diesel generator (${{\text{P}}}_{{\text{DG}}}^{{\text{new}}}$) is calculated by using the Lagrange multiplier method as shown in Eq. (23).

23 $$\left(\frac{1}{1-\frac{{{\text{dP}}}_{{\text{losses}}}}{{{\text{dP}}}_{{\text{DG}}}}} \right )\frac{{dC}_{DG-OP}}{{dP}_{DG}}= \frac{{d{ (C}_{MG-buy}\left(t\right) +{ C}_{MG-sell}(t))}}{{dP}_{grid(t)}}$$

∆P is the mismatch of power balance can be calculated as shown in Eq. (24), if it is power balance, the operating points of batteries and grid are kept constant**.**

24 $$\Delta {\text{P}}({\text{t}}) = {{\text{P}}}_{{\text{L}}}^{{\text{actual}}}({\text{t}})-{{\text{P}}}_{{\text{DG}}}^{{\text{new}}}\left({\text{t}}\right)-{{\text{P}}}_{{\text{PV}}}^{{\text{actual}}}\left({\text{t}}\right)-{{\text{P}}}_{{\text{WT}}}^{{\text{actual}}}\left({\text{t}}\right)-{\mathrm{P_{BESS}}}({\text{t}})-\mathrm{ P_{grid}}({\text{t}})$$

**Figure 5:**
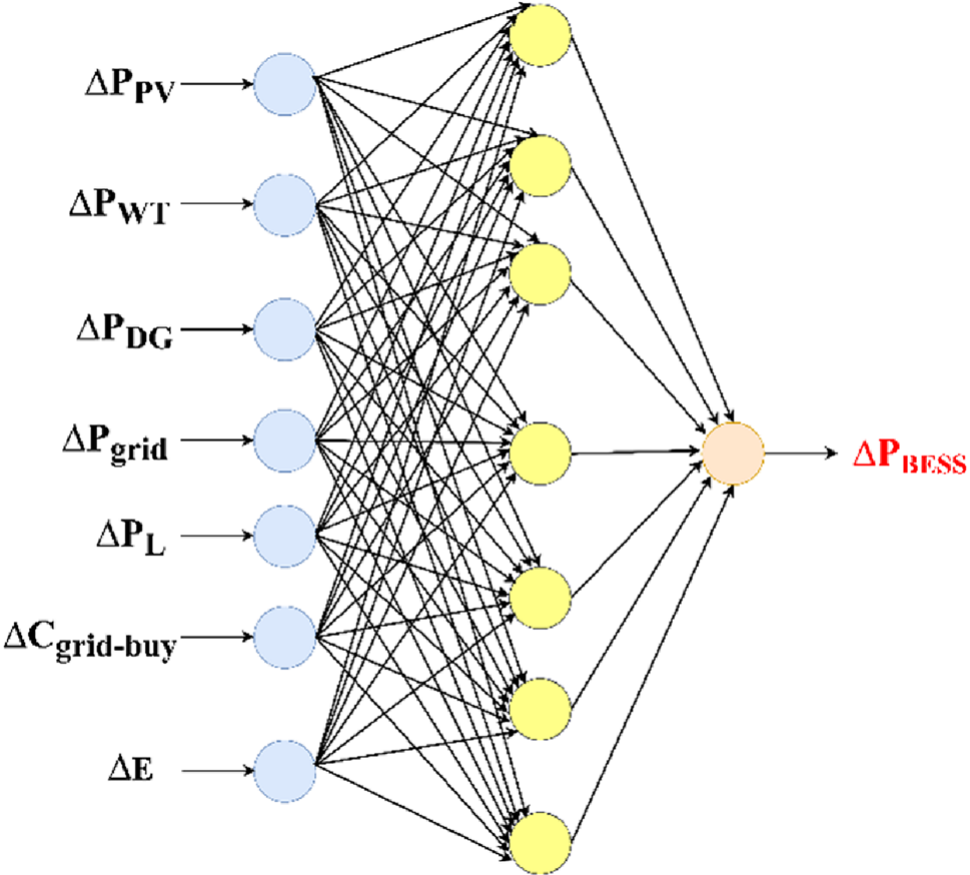
Structure of a neural network for real-time energy management system.

If ∆P is positive, it means the power demand is greater than the power output, so the set-point of the battery shall be increased in case of discharging or decreased in case of charging. Else if ∆P is negative, power demand is less than power output, so the set-point of the battery shall be decreased in case of discharging or increased in case of charging. The artificial neural network (ANN) is used to find ∆P_BESS_(t) which is determined by the input data of the ANN, the ANN for operation is not as complicated as the conventional EMS. The input data for the neural network is 7 variables: ∆P_PV_, ∆P_WT_, ∆P_grid_, ∆P_DG_, ∆P_L_, ${\Delta {\text{C}}}_{{\text{grid-buy}}}$ and ∆E as shown in Fig. 5.

The ∆P_PV_ (t), ∆P_WT_ (t), ∆P_DG_ (t), ∆P_grid_(t), ∆P_L_ (t), ∆C_grid-buy_ (t), and ∆E (t) can be calculated as follows:

25 $$\Delta \mathrm{P_{PV} }({\text{t}}) ={{\text{P}}}_{{\text{PV}}}^{{\text{actual}}}({\text{t}})-\mathrm{ P_{PV} }({\text{t}})$$

26 $$\Delta \mathrm{P_{WT} }({\text{t}}) ={{\text{P}}}_{{\text{WT}}}^{{\text{actual}}}({\text{t}})-\mathrm{ P_{WT }}({\text{t}})$$

27 $$\Delta \mathrm{P_{DG} }({\text{t}}) ={{\text{P}}}_{{\text{DG}}}^{{\text{new}}}({\text{t}})-\mathrm{ P_{DG} }({\text{t}})$$

28 $$\Delta \mathrm{P_{grid} }({\text{t}}) ={{\text{P}}}_{{\text{grid}}}^{{\text{new}}}({\text{t}})-\mathrm{ P_{grid} }({\text{t}})$$

29 $$\Delta \mathrm{P_{L} }({\text{t}}) ={{\text{P}}}_{{\text{L}}}^{{\text{actual}}}({\text{t}})-\mathrm{ P_{L} }({\text{t}})$$

30 $$\Delta {\text{C}}_{\text{grid-buy }}({\text{t}}) ={{\text{C}}}_{{\text{grid-buy}}}^{{\text{actual}}}({\text{t}})-\mathrm{ C}_{\text{grid-buy }}({\text{t}})$$

31 $$ \Delta {\text{E }}\left( {\text{t}} \right) \, = {\text{E}}_{{{\text{new}}}} \left( {{\text{t}} + {1}} \right) \, {-}{\text{ E}}_{{}} \left( {\text{t}} \right) $$

where: ${{\text{P}}}_{{\text{PV}}}^{{\text{actual}}}$, ${{\text{P}}}_{{\text{WT}}}^{{\text{actual}}}$, ${{\text{P}}}_{{\text{DG}}}^{{\text{new}}}$_,_ ${{\text{P}}}_{{\text{grid}}}^{{\text{new}}}$, ${{\text{P}}}_{{\text{L}}}^{{\text{actual}}}$, ${{\text{C}}}_{{\text{grid}}-{\text{buy}}}^{{\text{actual}}}$ and E_new_ are the actual output power of the PV, actual output power of the WT, new setpoint of DG, new setpoint of main grid, actual load demand, actual purchased electricity cost, and the new energy stored in the battery, respectively.

The value of ∆P_BESS_(t) must be subject to the following constraints:

32 $${{-{\text{P}}}_{{\text{BESS}},{\text{max}}}\le ({\text{P}}}_{{\text{BESS}}}\left({\text{t}}\right)+ \Delta {{\text{P}}}_{{\text{BESS}}}({\text{t}}))\le {{\text{P}}}_{{\text{BESS}},{\text{max}}}$$

33 $$ {\text{E}}_{{{\text{min}}}} < \, ({\text{E}}\left( {{\text{t}} + {1}} \right) \, + (\Delta {\text{T }} \times \Delta {\text{P}}_{{{\text{BESS}}}} \left( {\text{t}} \right))) \, < {\text{ E}}_{{{\text{max}}}} $$

where, E_min_ and E_max_ are the allowable minimum and maximum stored energy in the battery, respectively.

The new set-point of battery (${{\text{P}}}_{{\text{BESS}}}^{{\text{new}}}$ (t)) is calculated as shown in Eq. (34).

34 $${{\text{P}}}_{{\text{BESS}}}^{{\text{new}}}({\text{t}})\hspace{0.17em}=\hspace{0.17em}\mathrm{P_{BESS }}({\text{t}})\hspace{0.17em}+\hspace{0.17em}\Delta \mathrm{P_{BESS }}({\text{t}})$$

The new total active power balance equation in the MG is calculated as follows:

35 $${{\text{P}}}_{{\text{grid}}}^{{\text{new}}}({\text{t}})={{\text{P}}}_{{\text{L}}}^{{\text{actual}}}({\text{t}})-{{\text{P}}}_{{\text{DG}}}^{{\text{new}}}({\text{t}})-{{\text{P}}}_{{\text{PV}}}^{{\text{actual}}}({\text{t}})-{{\text{P}}}_{{\text{WT}}}^{{\text{actual}}} ({\text{t}})-{{\text{P}}}_{{\text{BESS}}}^{{\text{new}}}({\text{t}})$$

**Figure 6:**
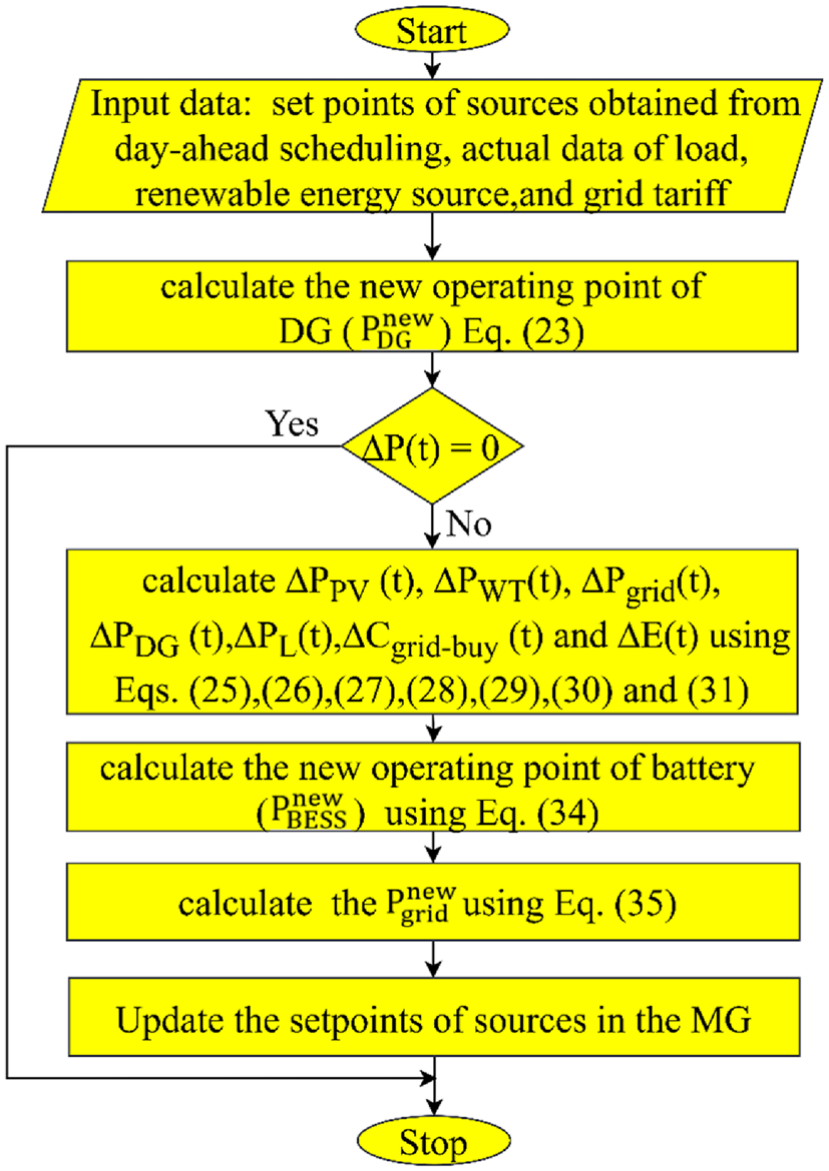
Flow chart of the proposed real-time energy management system.

The flow chart of the proposed real-time energy management system for economic dispatch during real-time can be summarized in Fig. 6.

## Simulation results

In this section, the performance of the proposed bi-level EMS is investigated and evaluated.

### Day-ahead scheduling (Level I) results

The day-ahead scheduling of the MG's generation units is determined by the day-ahead forecasting of weather and load demand.

#### Day-ahead forecasting (stage I)

**Figure 7:**
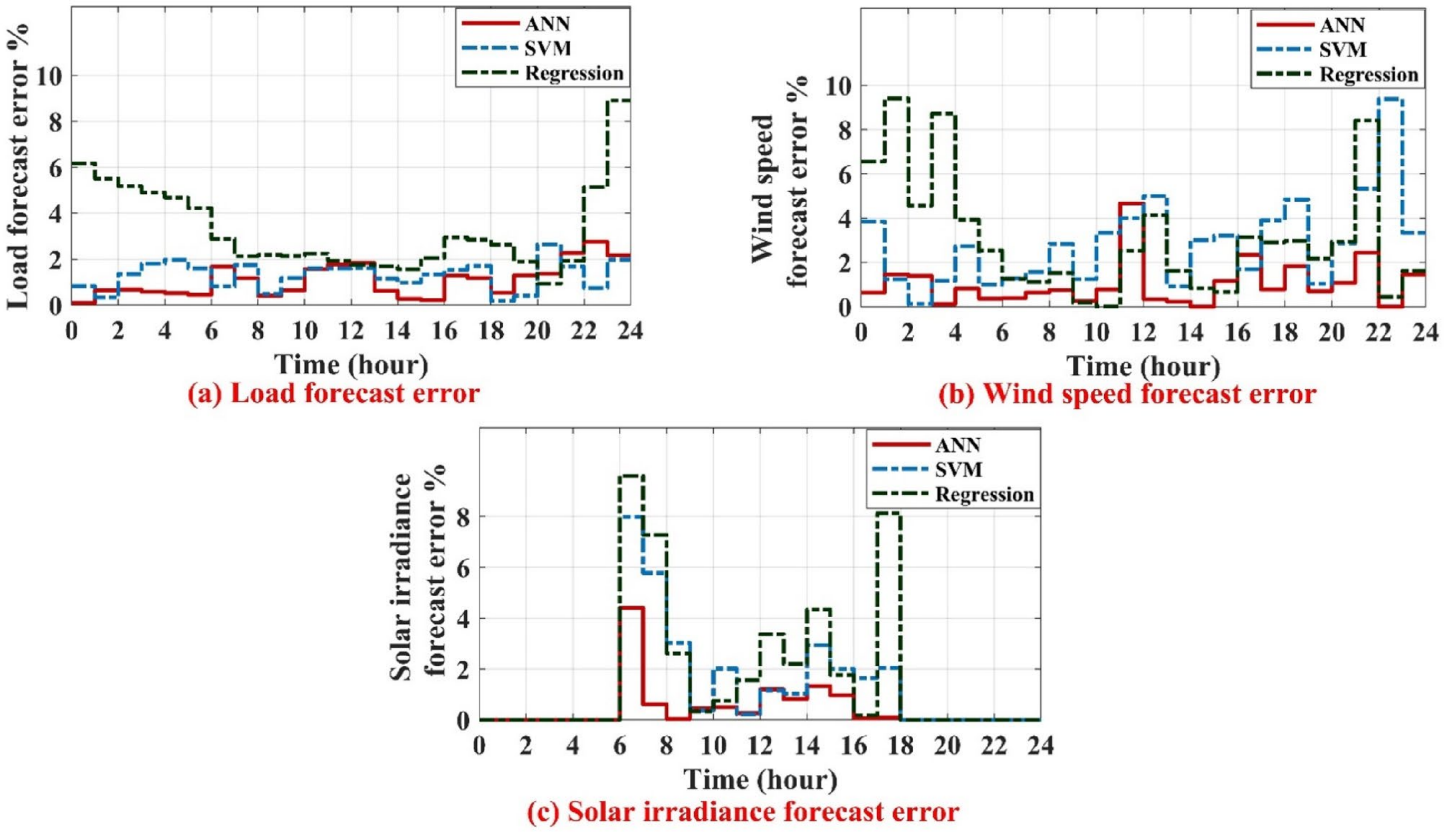
Forecast error of load, wind speed and solar irradiance.

For the regression based forecasting, the MAPE of load and wind speed forecasting is 3.26% and 3.086%, respectively. While for the SVM based forecasting, the MAPE is 1.302% and 2.868% for load and wind speed forecasting, respectively. But for the ANN based forecasting, the MAPE is 1.08% and 1.025% for load and wind speed forecasting, respectively. The RMSE of solar irradiance is 13.66 W/m^2^, 8.77 W/m^2^ and 3.9 W/m^2^ for regression, SVM and ANN based forecasting, respectively. ANN provides less forecasting error of load, wind speed and solar irradiance as compared to the forecasting techniques that are based on Support Vector Machine (SVM) and traditional technique, regression method, as shown in Fig. 7a–c, respectively.

It can be concluded that, ANN based short term forecasting is more accurate than regression based approach and SVM based approach as far as MAPE and RMSE are considered as shown in Table 2.

**Table 2:** Comparison of performance short term forecasting methods.

	Regression based approach	Support vector machine (SVM)	Artificial neural networks (ANN)
MAPE of load (%)	3.26	1.302	1.08
RMSE (kW) of load	117.87	48.57	43.97
RMSE (W/m^2^) of solar irradiance	13.66	8.77	3.9
MAPE of wind speed (%)	3.086	2.868	1.025
RMSE (m/s) of wind speed	0.318	0.228	0.098

#### Day-ahead unit commitment (stage II)

**Figure 8:**
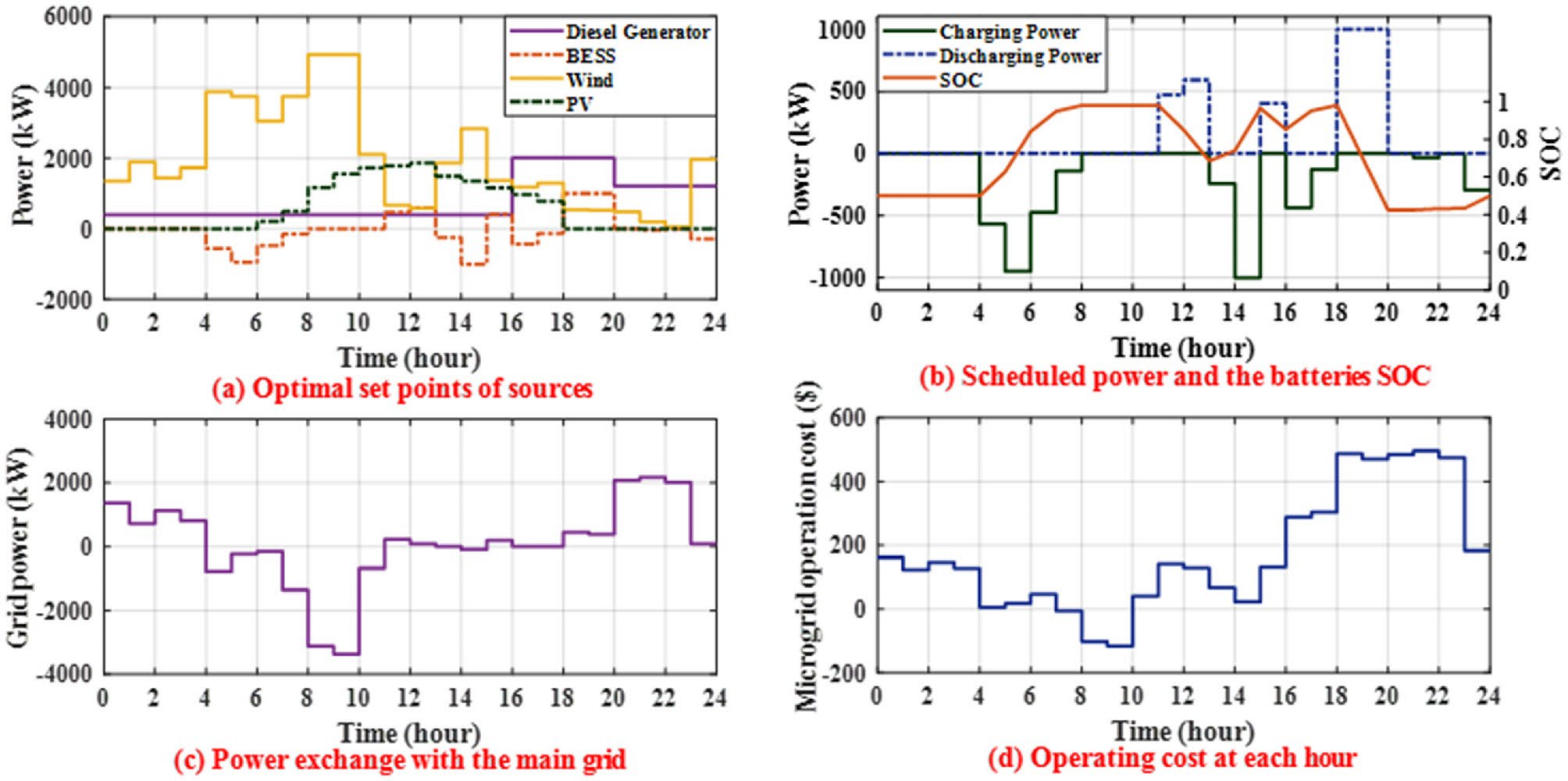
Results obtained by CHIO.

At 12 midnight, the initial SOC of the batteries is assumed to be 50%. The optimal set-points of MG sources obtained by CHIO for the day ahead are shown in Fig. 8a. Figure 8b depicts the optimal day-ahead set-points of batteries and their SOC as determined by CHIO. The power exchanged with the main grid to maintain system balance is shown in Fig. 8c, where a positive indicates that the MG imports power from the main grid and a negative indicates that the MG exports power to the main grid.

At 2 a.m., the load at MG (2955.8 kW) requires more power than can be generated by PV (0 kW), WT (1433.2 kW), and DG (400 kW), so the battery is expected to operate in discharging mode and provide power to cover the rest load for the economic dispatch at 2 a.m. However, for CHIO-based day-ahead scheduling, the battery will be operated in idle mode, as shown in Fig. 8b. Although it may be optimum to discharge the battery to cover the excess load at 2 a.m., the optimization algorithm prevents battery discharging and imports more power from the main grid, as battery discharging is not the optimal option when considering the daily operating cost. At 12 p.m., the electricity generated by PV (1858.8 Kw), WT (582.5 Kw), and DG (400 Kw) is less than the load at MG (3514 Kw). While the battery is in discharging mode, it will produce roughly 590.4 kW, or 59.04% of its rated power. According to CHIO, as shown in Fig. 8c, the MG imports roughly 82.3 kW from the main grid to cover the remaining MG load. At 9 p.m., the electricity generated by PV (0 Kw), WT (198.4 Kw), and DG (1212.7 Kw) is less than the load at MG (3549.5 Kw). While the battery is in charging mode and draws about 35.4 kW which represents 3.54% of its rated power and its SOC reaches about 43.2% after 1 h as shown in Fig. 8b, the MG imports roughly 2173.8 kW from the main grid to cover the remaining MG load. For the CHIO, the battery will run for approximately 11 h in charging mode, 5 h in discharging mode, and 8 h in idle mode. The battery SOC is reset by the algorithm to 50% at midnight on the last day in order to maintain the initial SOC and allow for charging or discharging at the start of the day. Figure 8d displays the energy consumption cost of the MG due to CHIO for each hour of the next day. The total cost of MG operation in the next 24-h obtained by CHIO will be $ 4137.

### Real-time scheduling (level II) results

**Figure 9:**
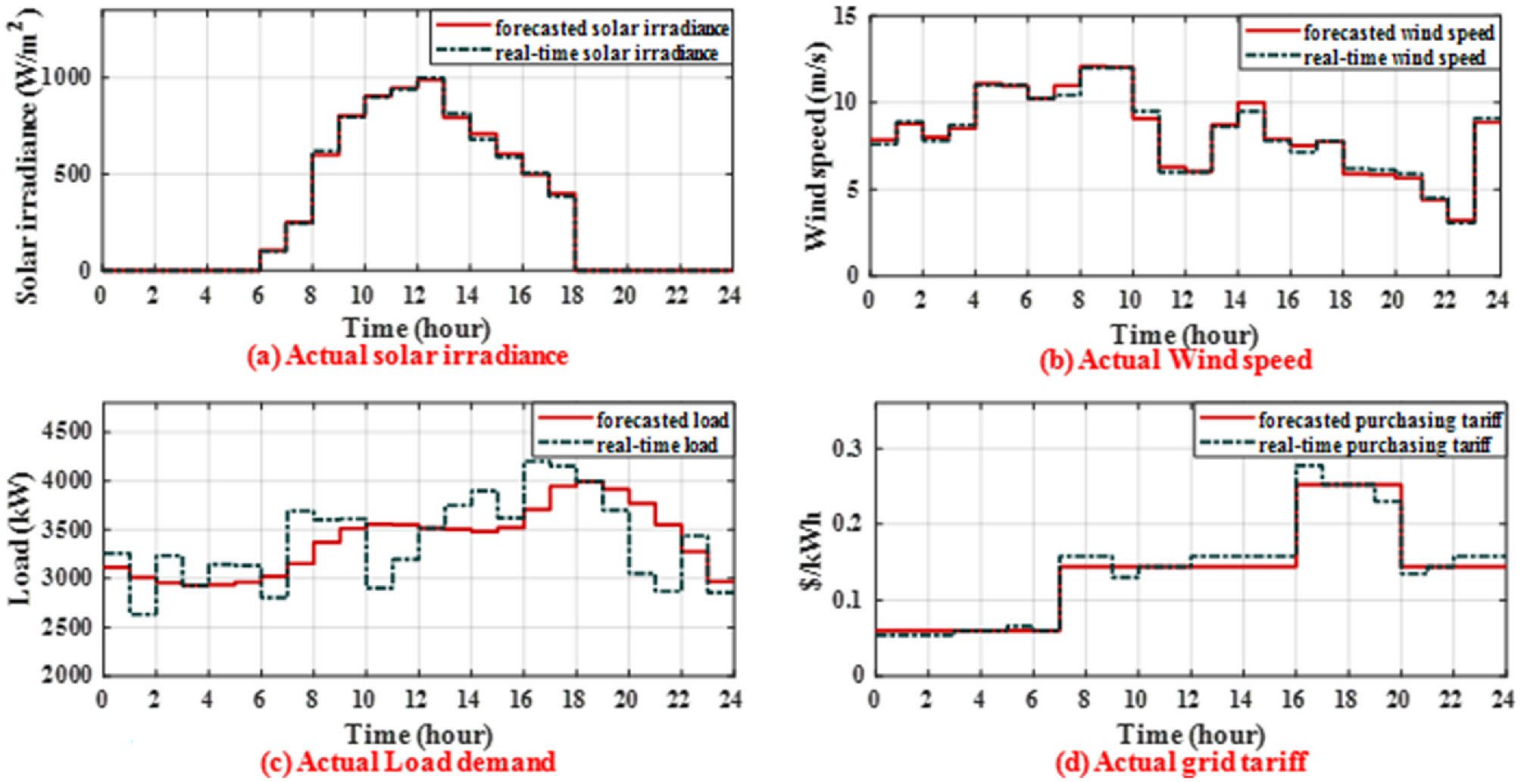
The forecasted and actual data of solar irradiance, wind speed, load demand and grid tariff.

**Figure 10:**
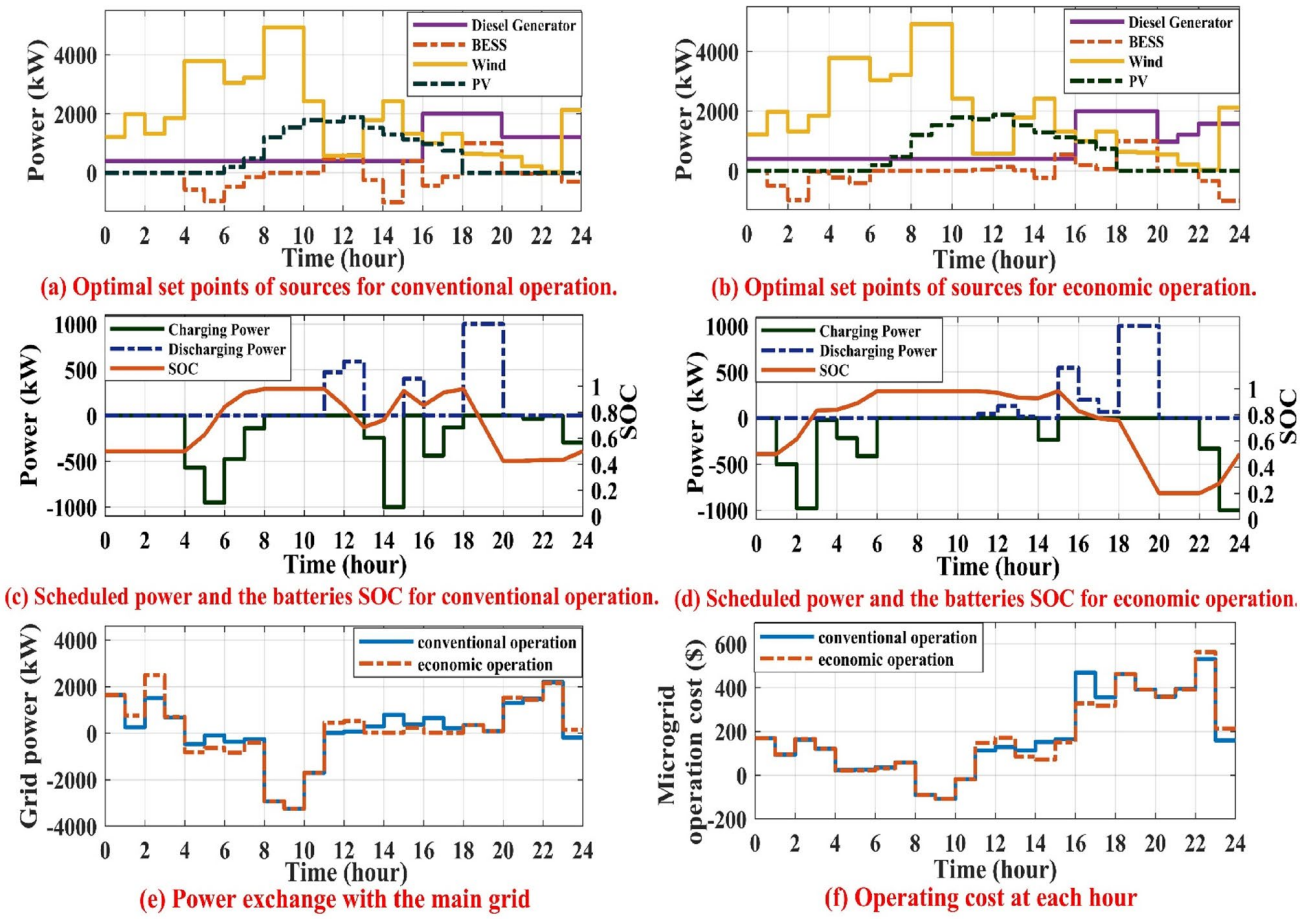
Results obtained by real-time conventional and economic operations.

This scenario involves some deviations from the expected values of solar irradiance, wind speed, grid tariff, and load in order to assess how well the suggested energy management strategy performs in real-time under uncertain conditions to achieve economic operation while maintaining system balance. The solar irradiance, wind speed, load, and grid tariff uncertainties and actual data are shown in Fig. 9a–d. The solar irradiance, wind speed, and load forecasted are generated from the day-ahead forecasting stage based on ANN models, but the forecasted grid tariff is from Table 1. The actual data is generated by adding bounded uncertainties that may occur during real time on the forecasted data. Figure 10 displays the results of the real-time conventional and economic operations. Figure 10a displays the optimal set-points of MG sources for real time conventional operation while Fig. 10b displays the updated optimal set-points for the sources for real time economic operation. Figure 10c displays the optimal set-points for batteries along with their SOC in real-time conventional operation while Fig. 10d displays the new optimal set-points for batteries along with their SOC in real-time economic operation. Figure 10e displays the power exchanged with the main grid during real-time. Figure 10f displays the MG operating cost for each hour of the day. In conventional real-time operation, the grid maintains system balance and does not update the set-points of batteries and DG in real-time. On the other hand, to achieve real-time economic operation, the DG and battery set-points will be rescheduled in real-time.

At 3 a.m., the output power from WT is increased by 121.4 kW about the prescheduled value, while the actual load demand is kept constant. For real-time economic operation, the operating points of batteries and grid are updated to -23.4 kW(charging mode) and 705.4 kW, respectively, to cover the load with less cost during real-time operation, while for real-time conventional operation, the set-points of DG and batteries are kept at the values obtained from day-ahead scheduling and the power drawn from the main grid decreased to 682 kW, as shown in Table 3. At 11 a.m, the actual wind speed is 6 m/s (4.45% less than the expected value), the actual solar irradiance is 940 W/m^2^ (0.78% less than the expected value), and the actual load demand is 3195.6 kW (about 9.92% less than the expected value). As a result, the power generated from WT has decreased from 667.4 to 576.3 kW, and the power provided by PV has decreased from 1782.5 to 1730.8 kW. For real-time economic operation, the operating points of batteries and grid are updated to 45.6 kW (Discharging mode) and 442.9 kW, respectively, to achieve economic operation under uncertainties in solar irradiance, wind speed, and load. But for real-time conventional operation, the power drawn from the main grid is decreased to 17 kW to keep the system balanced, as shown in Table 3.

**Table 3:** Optimal set-points of sources, SOC of battery and exchanged power with the grid at 3 a.m., 11 a.m., 5 p.m. and 11 p.m.

EMS level	Time (h)	P_PV_ (kW)	P_WT_ (kW)	P_DG_ (kW)	P_BESS_ (kW)	P_grid_ (kW)	P_L_ (kW)	SOC	Cost ($)
Day-ahead scheduling	3 a.m.:4 a.m.	0	1727.6	400	0	803.4	2931	0.5:0. 5	4137
	11 a.m.:12 p.m.	1782.5	667.4	400	471.5 (discharging mode)	226.3	3547.7	0.98:0.849	
	5 p.m.:6 p.m.	775.5	1299	2000	− 129.4 (charging mode)	0	3945.1	0.95:0.98	
	11 p.m.:12 a.m.	0	1962.2	1212.7	− 294.8 (charging mode)	88.1	2968.2	0.433 :0.5	
Real-time conventional operation	3 a.m. :4 a.m.	0	1849	400	0	682	2931	0.5:0. 5	4266.2
	11 a.m.:12 p.m.	1730.8	576.3	400	471.5 (discharging mode)	17	3195.6	0.98:0.849	
	5 p.m.: 6 p.m.	747.1	1319.9	2000	− 129.4 (charging mode)	212.4	4150	0.95:0.98	
	11 p.m.:12 a.m.	0	2122.4	1212.7	− 294.8 (charging mode)	− 184.6	2855.7	0.433 :0.5	
Real-time economic operation	3 a.m.:4 a.m.	0	1849	400	− 23.4 (charging mode)	705.4	2931	0.832 :0.838	4118.7
	11 a.m.:12 p.m.	1730.8	576.3	400	45.6 (discharging mode)	442.9	3195.6	0.98:0.967	
	5 p.m.:6 p.m.	747.1	1319.9	2000	65.6 (discharging mode)	17.4	4150	0.773:0.755	
	11 p.m.:12 a.m.	0	2122.4	1581.8	− 1000 (charging mode)	151.5	2855.7	0.275:0.5	

At 5 p.m., the output power from WT is increased by 20.9 kW about the prescheduled value, while the actual load demand is 4150 kW (about 5.19% more than the expected value). For real-time economic operation, the operating points of batteries and grid are updated to 65.6 kW (discharging mode) and 17.4 kW, respectively, to cover the load with less cost during real-time operation, while for real-time conventional operation, the set-points of DG and batteries are kept at the values obtained from day-ahead scheduling and the imported power from the main grid is increased to 212.4 kW, as shown in Table 3.

At 11 p.m., the actual wind speed is 9.1 m/s (2.59% more than the expected value), the grid tariff is 0.158 $/kWh (9.7% more than the expected value) and the actual load demand is 2855.7 kW (about 3.79% less than the expected value). As a result, the power generated from WT is increased from 1962.2 to 2122.4 kW. For real-time economic operation, the operating points of DG, batteries and grid are updated to 1581.8 kW, − 1000 kW (Charging mode) and 151.5 kW, respectively, to cover the load with less cost during real-time operation, while for real-time conventional operation, the set-points of DG and batteries are kept at the values obtained from day-ahead scheduling and the export power to the main grid is increased to 184.6 kW to keep the system balanced, as shown in Table 3. Real-time optimal scheduling minimizes the MG operating cost through the day from $ 4266.2 to $ 4118.7, saving about 3.46% of the total cost compared to real-time conventional operation.

## Conclusion

An optimal energy management strategy based on two levels, day-ahead scheduling and real-time scheduling, for a grid tied microgrid with the aim of minimizing the operational cost while satisfying the different technical constraints is proposed. Also, the efficient day-ahead scheduling is based on two stages, day-ahead forecasting and day-ahead unit commitment, for optimal scheduling of the sources in the MG through the next 24-h.

The obtained simulation results show that the ANN based day-ahead forecasting more accurate predictions of the solar irradiance, wind speed and load for the next day as compared to the forecasting techniques that are based on support vector machine and traditional technique which is regression-based forecasting. The daily operating cost of MG is $4137 for the ideal day-ahead scheduling. The real-time scheduling, 2nd level of the proposed EMS, saves about 147.5 $ per day (about 3.46% of the total operating cost is reduced) by updating the set points of MG sources according to the actual data of solar irradiance, wind speed, load and grid tariff during real-time operation.

In conclusion, the suggested bi-level EMS is an effective approach for improving the microgrid performance and reducing the energy consumption costs.

## Supplementary Information

Supplementary Tables.

## Data Availability

All data generated or analyzed during this study are included in this published article and its supplementary information files.

## Appendix

### System parameters^6,38^

**Table:**

Parameter	Value	Parameter	Value
$$\mathrm{\alpha }$$	0.005 W/C	SOC_min_	20%
T_r_	25 C	SOC_max_	98%
P_nom_	5000 Kw	B_capacity_	4000 kWh
V_ci_	2.5 m/s	C_deg_	$${1.2 \times 10}^{-9}$$$/W
V_r_	12 m/s	N_cycle_	4000
V_co_	25 m/s	CC_bat_	456 $/kWh
∆T	1 h	$${\eta }_{c{\text{h}}}$$	90%
P_DG,min_	400 Kw	$${\eta }_{d{\text{is}}}$$	90%
P_DG,max_	2000 Kw	$$\mathrm{\alpha }$$_DG_	38.16 ($/h)
∆T	1 h	$$\upbeta $$_DG_	0.09799 $/kWh
SOC_ini_	50%	$$\upgamma $$_DG_	$${1.896*10 }^{-5}$$ ($/kWh)^2^
