# Supplementary material for: Multi-level optimal energy management strategy for a grid tied microgrid considering uncertainty in weather conditions and load
Source: Sci Rep. 2024 May 2;14:10059. doi: 10.1038/s41598-024-59655-7 (PMC11551214; doi:10.1038/s41598-024-59655-7)
Supplement: Supplementary file 1 — Supplementary Tables. [file 41598_2024_59655_MOESM1_ESM.pdf]

Supplementary Table 1. (Purchasing and selling electricity tariffs[1])

Type	Time	Value
**Off-peak purchasing tariff**	From 12 am to 7 am	0.06 $/kWh
**Mid-peak purchasing tariff**	From 7 am to 4 pm	0.144 $/kWh
**Peak purchasing tariff**	From 4 pm to 8 pm	0.252 $/kWh
**Mid-peak purchasing tariff**	From 8 pm to 12 am	0.144 $/kWh
**Fixed selling tariff**	All day	0.0582 $/kWh

Supplementary Table 2. (System Parameters[1,2])

Parameter	Value	Parameter	Value
**α**	0.005 W/C	SOC_{min}	20%
T_r	25 C	SOC_{max}	98%
P_{nom}	5000 Kw	$B_{capacity}$	4000 kWh
V_{ci}	2.5 m/s	C_{deg}	1.2 x 10^{-9} $/W
V_r	12 m/s	N_{cycle}	4000
V_{co}	25 m/s	CC_{bat}	456 $/kWh
ΔT	1 hour	η_{ch}	90%
$P_{DG,min}$	400 Kw	η_{dis}	90%
$P_{DG,max}$	2000 Kw	α_{DG}	38.16 ($ /h)
ΔT	1 hour	β_{DG}	0.09799 $ /kWh
SOC_{ini}	50%	γ_{DG}	$1.896 * 10^{-5}$ ($/kWh)^2

References

1. Elkazaz, M., Sumner, M. & Thomas, D. Energy management system for hybrid PV-wind-battery microgrid using convex programming, model predictive and rolling horizon predictive control with experimental validation. *Int. J. Electr. Power Energy Syst.* **115**, 105483 (2020).
2. Taha, M. S., Abdeltawab, H. H. & Mohamed, Y. A.-R. I. An online energy management system for a grid-connected hybrid energy source. *IEEE J. Emerg. Sel. Top. Power Electron.* **6**, 2015–2030 (2018).
